# Schwann cells modified to secrete MANF is a potential cellular therapy for peripheral nerve regeneration

**DOI:** 10.1186/s13619-025-00247-9

**Published:** 2025-07-07

**Authors:** Bhadrapriya Sivakumar, Caleb Hammond, Valeria Martinez, Nickson Joseph, Johnson V. John, Anil Kumar, Anand Krishnan

**Affiliations:** 1 Department of Anatomy, Physiology, and Pharmacology, College of Medicine, University of Saskatchewan, 107 Wiggins Road, Saskatoon, SK S7N 5E5 Canada; 2 Cameco MS Neuroscience Research Centre, 701 Queen St, Saskatoon, SK S7K 0M7 Canada; 3 Department of Biochemistry, Microbiology, and Immunology, College of Medicine, University of Saskatchewan, 107 Wiggins Road, Saskatoon, SK S7N 5E5 Canada; 4 Terasaki Institute for Biomedical Innovation, 1018 Westwood Blvd, Los Angeles, CA 90024 USA

**Keywords:** MANF, Nerve regeneration, Schwann cell, Sensory neuron, Neurite outgrowth, Dorsal root ganglia, Nerve explant, Nerve repair, Cell plasticity, Peripheral nerves

## Abstract

Effective therapies for peripheral nerve repair are still lacking despite active research in this field over the past years. The limited knowledge of biomolecules that equally promote axon regeneration and glial cell dynamics, which are critical for nerve regeneration, poses a major challenge in developing effective therapies. Here, we showed that the neurotrophic factor mesencephalic astrocyte-derived neurotrophic factor (MANF) equally promotes axon regeneration and glial cell dynamics favorable for nerve regeneration. Using adult rodent models, we showed that the endogenous expression of MANF is restricted to non-peptidergic sensory neurons. However, supplementation of exogenous MANF promoted the growth of all subtypes of adult sensory neurons. We also demonstrated that exogenous MANF promotes the proliferation and migration of adult primary Schwann Cells (SCs). Furthermore, we showed that local and repeated administration of MANF to injured nerves promotes axon regeneration in mice models. Finally, we devised a therapeutic approach by programming nerve-resident SCs to locally and continuously deliver MANF to injured nerves and showed that this approach improves axon regeneration. Overall, this work developed a therapeutic approach by harnessing the power of SCs as a local delivery system for MANF for nerve repair.

**Supplementary Information:**

The online version contains supplementary material available at 10.1186/s13619-025-00247-9.

## Background

Peripheral nerve injuries are very common and account for significant clinical and socioeconomic burden worldwide (Bergmeister et al. 2020). However, currently available therapies are ineffective in completely repairing nerves and restoring functions. The lack of effective treatments often leads to permanent loss of functions in those who are met with severe nerve injuries. A primary hurdle to translating experimental therapies into clinical therapies is the challenge of maintaining their local availability in regenerating nerves (Alastra et al. 2021). The limited knowledge of biomolecules that can facilitate both axon regeneration and glial cell dynamics, which are critical for nerve regeneration, also hinders the development of effective therapies.

Peripheral nerve regeneration involves complex sequential events initiated by the clearance of axonal and myelin debris from the injured nerves by macrophages and Schwann Cells (SCs), the major glial cell population in peripheral nerves (Krishnan et al. 2024). The SCs then de-differentiate, proliferate, and migrate in an orderly manner toward the injured proximal nerve stump and direct the regenerating proximal axons toward target tissues (Krishnan et al. 2024). Clearing the debris is the most efficient process among these complex events. Although axon regeneration occurs initially, and may last for several weeks to months, it gradually diminishes because of the lack of local trophic support. SCs also gradually lose their dynamics because of chronic denervation and lack of support from axons (Fu and Gordon 1995). Hence, even after surgical correction, nerve regeneration often fails due to diminished growth response from axons and SCs. Therapies that improve and maintain both axon regeneration and SC dynamics when available locally to injured nerves should improve regenerative outcomes.

Recent work from our lab and others demonstrated that the neurotrophic factor mesencephalic astrocyte-derived neurotrophic factor (MANF) is a potential therapeutic candidate for nerve regeneration (Bautista et al. 2023; Lee et al. 2023). However, the endogenous availability of MANF in injured peripheral nerves has not been systematically explored. Similarly, whether MANF is equally effective in promoting axon regeneration and SC dynamics, which are critical for complete nerve regeneration, has not been systematically investigated. The efficacy of MANF to improve crush injuries, the most common form of nerve injury presented to neuro-clinics, has also not been examined. In this work, we demonstrated that MANF is preferentially expressed in a subtype of adult sensory neurons known as the small calibre non-peptidergic sensory neurons, indicating the requirement of exogenous MANF for large calibre neurons to ramp up their regeneration. We further found that supplementation of exogenous MANF improved the outgrowth of all subtypes of sensory neurons regardless of their endogenous MANF levels. Furthermore, we showed that direct and repeated delivery of MANF to injured nerves promotes axon regeneration in vivo, suggesting that repeated local supplementation of MANF is critical for effective nerve regeneration. Strikingly, we noted that MANF is only sparsely expressed in SCs, but they respond to external MANF with increased dynamics. Thus, we developed a potential therapeutic approach by harnessing SCs to serve as a local delivery system for MANF in injured nerves. We demonstrated that this approach effectively improved axon regeneration and is worth pursuing for therapy development.

## Results

### MANF is upregulated in injured DRGs and preferentially expressed in non-peptidergic sensory neurons

**Fig. 1:**
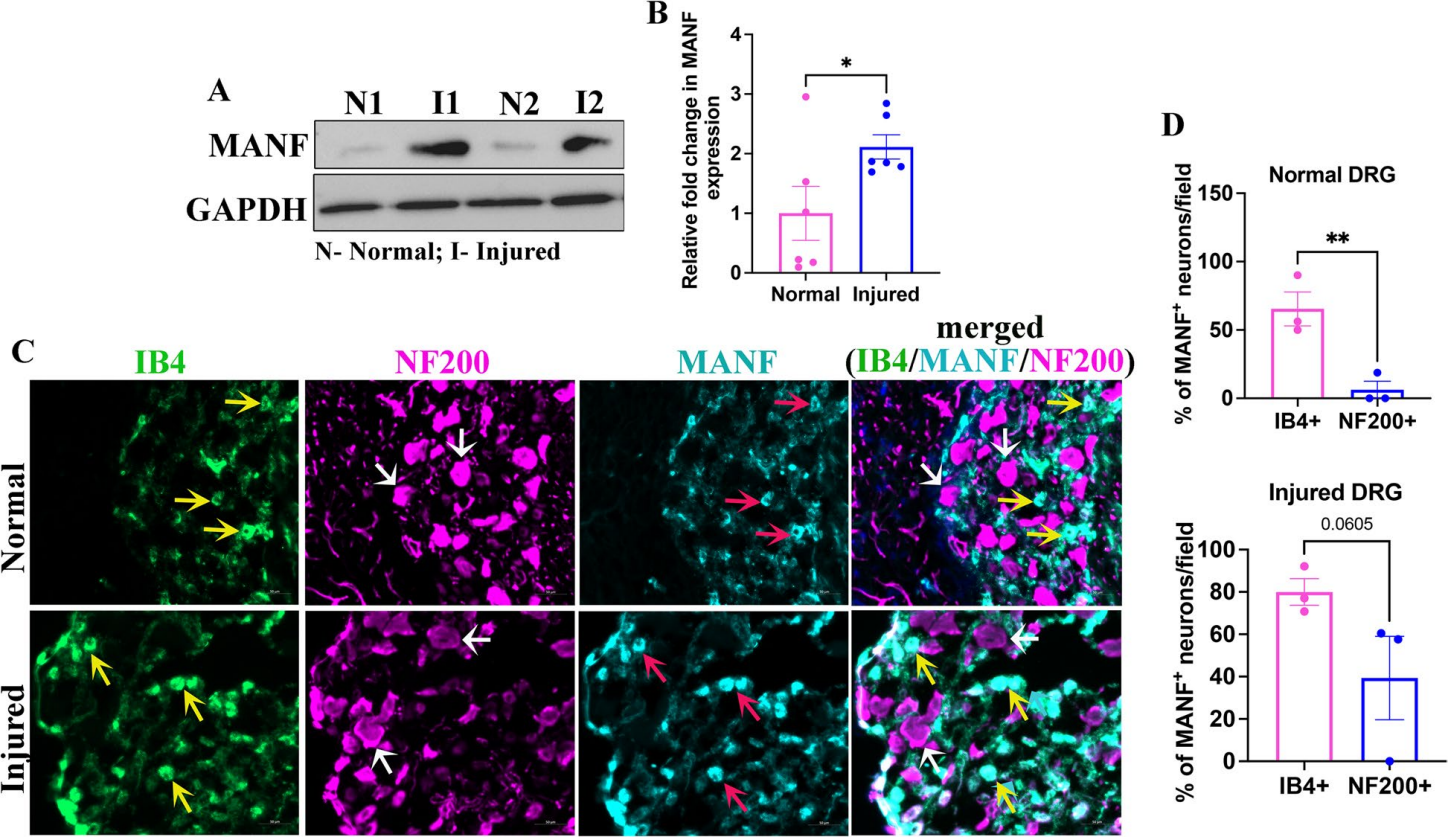
MANF is preferentially expressed in non-peptidergic sensory neurons in adult DRGs. **A** Western blot shows the expression of MANF in the normal and injured (3-day transection injury to sciatic nerve) adult rat DRGs. GAPDH is used as the loading control. **B** Quantification of MANF from western blot assay shows the induction of MANF in injured DRGs (data presented as mean ± SE; standard ‘t’ test; *n* = 6; *p < 0.05). **C** Co-immunostaining of IB4 (yellow arrows), NF200 (white arrows) and MANF (red arrows) in normal and injured DRGs from adult rats show the preferential expression of MANF in IB4 positive non-peptidergic sensory neurons (yellow arrows in the merged view) (scale bar, 50 µm). **D** Quantification of percentage of MANF expressing IB4^+^ and NF200^+^ neurons in normal (top) and injured (bottom) adult rat DRGs show the preferential expression of MANF in IB4 positive non-peptidergic sensory neurons (data presented as mean ± SE; standard ‘t’ test; *n* = 3; **p < 0.01)

Our recent proteomics studies using LC-MS/MS have shown that MANF is upregulated in growth-primed DRGs (Bautista et al. 2023). To validate this observation, we examined the expression of MANF in non-injured and injured adult DRGs *(ipsilateral DRGs from adult SD rats after 3 days of sciatic nerve transection)* using western blot and found that it is significantly upregulated in injured DRGs (Fig. 1A, B). DRGs are primarily composed of several subtypes of sensory neurons and satellite glial cells. We previously showed that MANF is preferentially expressed in a subtype of sensory neurons that poorly express NF200 (NF200^poor^ neurons) (Bautista et al. 2023). To further characterize the identity of MANF-expressing neurons, we performed co-immunostaining experiments. NF200^high^ neurons are large calibre neurons that project myelinating axons, whereas Isolectin B4 (IB4) binding neurons (IB4^+^ neurons) are small calibre non-peptidergic neurons that project nociceptors. Co-immunostaining of NF200, IB4, and MANF in non-injured and injured DRGs showed that MANF is preferentially expressed in IB4^+^ neurons (Fig. 1C, D). It is thus likely that the upregulation of MANF in injured DRGs is a mere reflection of its upregulation in IB4^+^ neurons. This indeed suggests that intrinsically MANF-deficient NF200^high^ neurons may require external MANF support for efficient regeneration.

### MANF expression is not altered in injured peripheral nerve

**Fig. 2:**
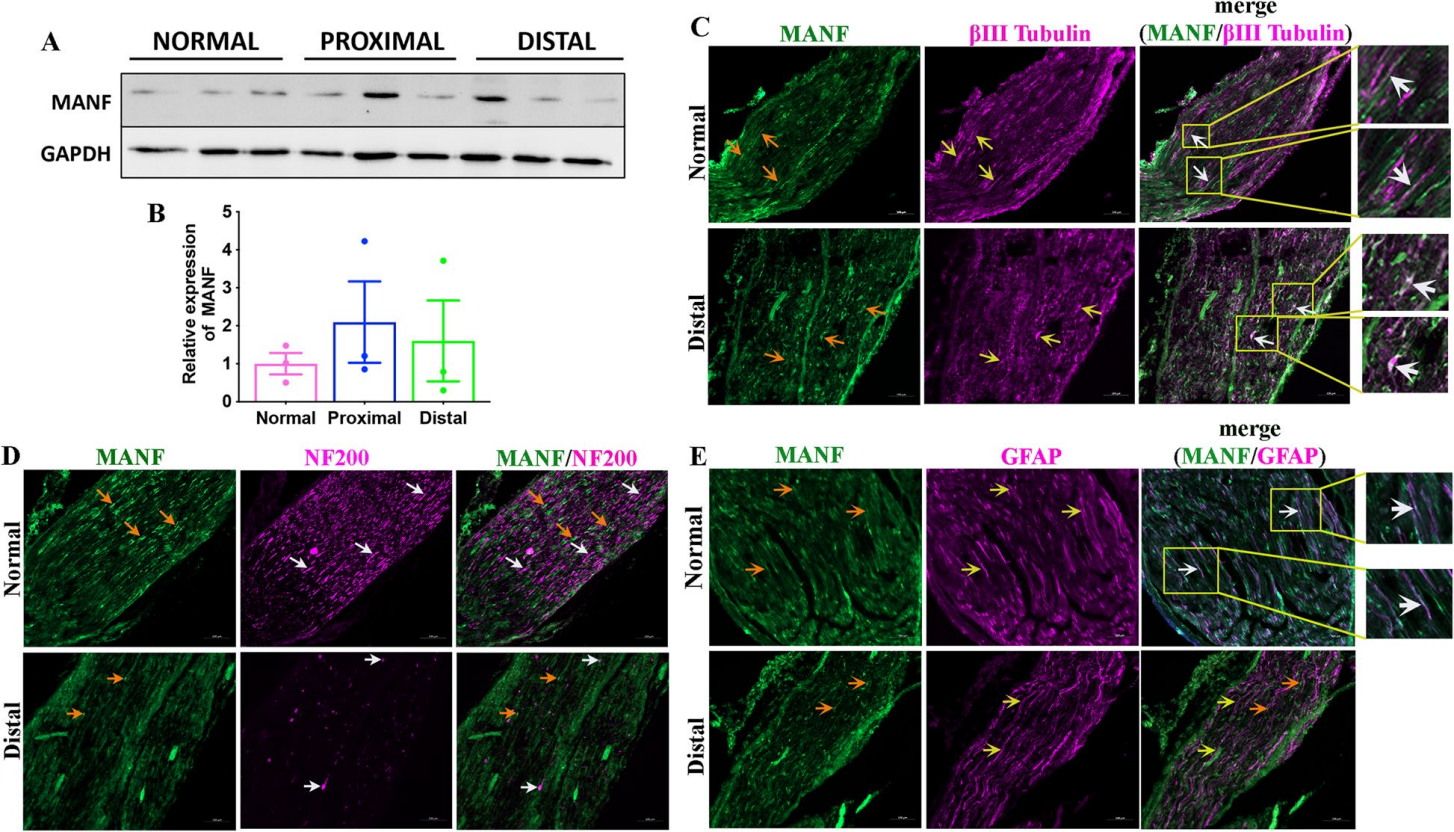
MANF expression does not change in injured peripheral nerves. **A** Western blot shows the expression of MANF in normal and injured (3-day transection injury) proximal and distal sciatic nerve segments of adult rats. GAPDH is used as the loading control. **B** Quantification of ‘A’ shows no significant change in the expression of MANF in injured nerves compared to the control (data presented as mean ± SE; One-Way ANOVA; *n* = 3). **C** Co-immunostaining of βIII tubulin (yellow arrows) and MANF (orange arrows) in normal and injured distal sciatic nerve of adult rat shows MANF expression in a subpopulation of βIII tubulin positive axons (white arrows in the merge image and enlarged insets) (scale bar, 100 µm). **D** Co-immunostaining of NF200 (white arrows) and MANF (orange arrows) in normal and injured distal sciatic nerve of adult rats shows no major co-localization of MANF with NF200 positive axons (scale bar, 100 µm). **E** Co-immunostaining of GFAP (yellow arrows) and MANF (orange arrows) in normal and injured distal sciatic nerve of adult rat shows sparing expression of MANF in SCs in the normal nerve (white arrows in the merge image and enlarged insets) (scale bar, 100 µm)

We next examined the expression of MANF in sciatic nerves to evaluate if MANF is upregulated in nerves after injury. Western blot experiments in non-injured control and injured proximal and distal sciatic nerve segments showed no significant difference in MANF expression, indicating no local induction of MANF in injured nerves (Fig. 2A, B). Although injury did not induce MANF, a low basal expression of MANF was apparent in both control and injured nerves. Therefore, we next examined the cellular distribution of MANF in nerves by co-immunostaining MANF with βIII tubulin, a pan-neuronal marker, NF200, a large calibre neuron marker, and GFAP, a SC marker. We found that MANF co-localizes with βIII tubulin^+^ axons (Fig. 2C). βIII tubulin marks both small and large calibre axons. However, we found no notable co-localization of MANF with NF200^+^ axons, which comprise large calibre axons, indicating that MANF is preferentially expressed in NF200^poor^ small calibre axons (Fig. 2D). Our immunostaining results showed co-localization of MANF and GFAP only at scattered locations, indicating that SCs express MANF only sparingly in both normal and injured peripheral nerves (Fig. 2E).

### Exogenous MANF promotes the outgrowth of adult primary sensory neurons in vitro regardless of their endogenous MANF expression profile

**Fig. 3:**
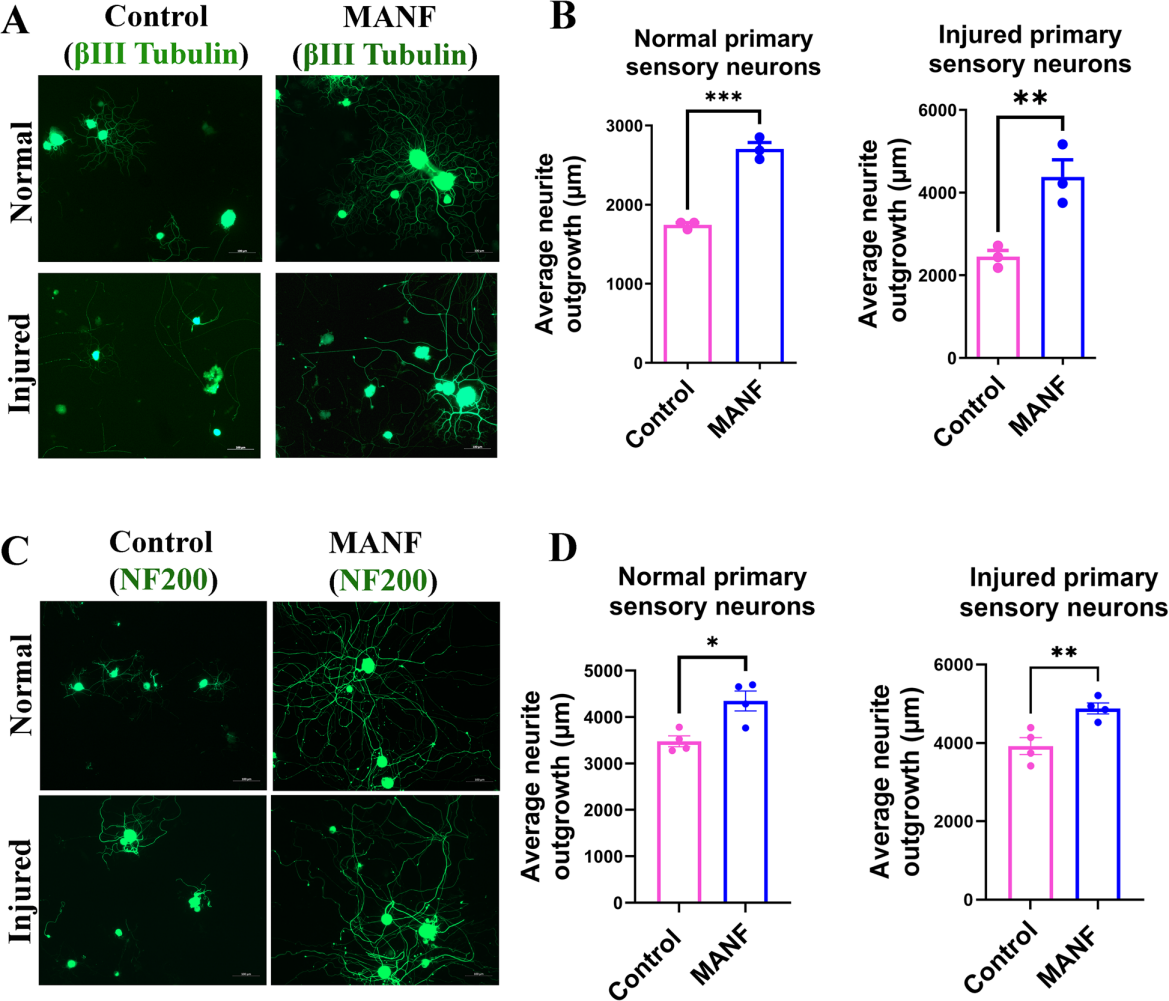
Exogenous MANF promotes the outgrowth of both normal and injured adult neurons in vitro. **A** βIII tubulin staining shows that supplementation of 100 and 50 ng/ml MANF promotes the outgrowth of normal and injured adult primary sensory neurons, respectively (scale bar, 100 µm). **B** Quantification of neurite outgrowth using WIS-NeuroMath Software shows significant induction of neurite outgrowth in both normal and injured βIII tubulin positive neurons after MANF supplementation (data presented as mean ± SE; Standard ‘t’ test; *n* = 3; **p < 0.01, ***p < 0.001). **C** NF200 staining shows that supplementation of 100 and 50 ng/ml MANF promotes the outgrowth of normal and injured adult primary sensory neurons, respectively (scale bar, 100 µm). **D** Quantification of neurite outgrowth using WIS-NeuroMath Software shows significant induction of neurite outgrowth in both normal and injured NF200 positive neurons after MANF supplementation (data presented as mean ± SE; Standard ‘t’ test; *n* = 4; *p < 0.05, **p < 0.01)

Our observation of the lack of induction of MANF in injured nerves indicates that, although injury induces MANF in DRGs, it is not efficiently transported to injured nerve terminals to support axon regeneration. Hence, supplementing exogenous MANF to injured nerves may promote nerve regeneration. Substantiating this argument, we previously showed that exogenous MANF promotes the outgrowth of normal NF200^high^ adult primary sensory neurons in vitro (Bautista et al. 2023). Here, we investigated if exogenous MANF is even effective in promoting the outgrowth of injured neurons to evaluate its potential as a therapy for nerve repair. We also examined if exogenous MANF promotes the outgrowth of all subtypes of neurons by evaluating neurite outgrowth after βIII tubulin staining, which stains both small (NF200^poor^/MANF^high^) and large (NF200^high^/MANF^low^) calibre neurons. We supplemented 100 and 50 ng/ml recombinant human MANF to the cultures of normal and injured adult rat primary sensory neurons, respectively. These doses for normal and injured neurons were determined based on our preliminary dose-dependent experiments, which showed increased growth response by normal and injured neurons at 100 and 50 ng/ml MANF, respectively. We found that exogenous MANF significantly promoted the outgrowth of both normal and injured primary sensory neurons regardless of the neuron subtypes, as both βIII tubulin^+^ and NF200^+^ neurons showed increased outgrowth after MANF supplementation (Fig. 3). Overall, these findings substantiate that MANF is a potential therapeutic candidate for peripheral nerve regeneration, requiring systematic therapeutic profiling.

### Exogenous MANF promotes SC proliferation and migration

**Fig. 4:**
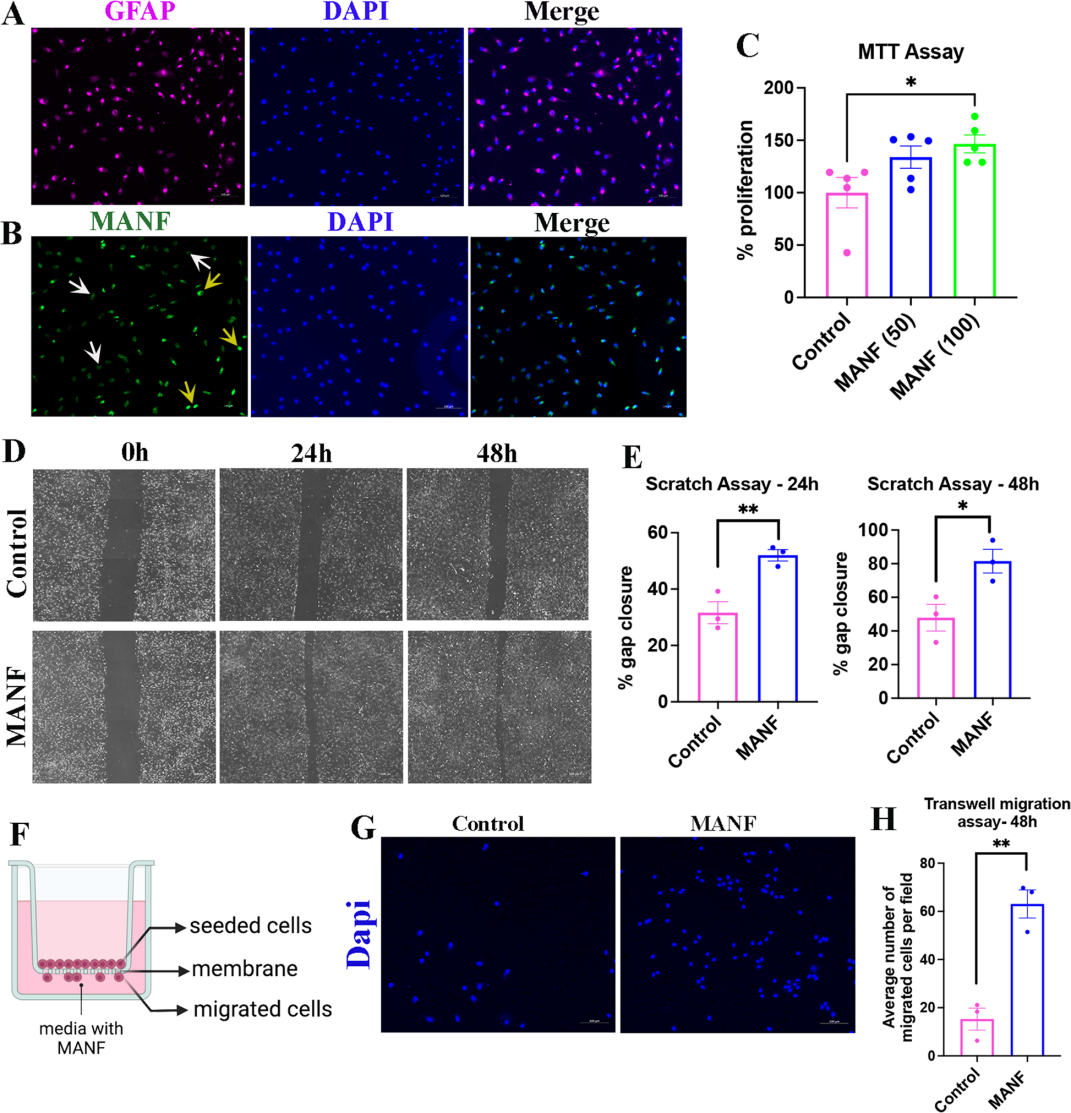
Exogenous MANF promotes SC dynamics. **A** Immunostaining of GFAP in adult rat primary SCs (scale bar, 100 µm). **B** Immunostaining of MANF in adult rat primary SCs. Yellow arrows show intense expression, and white arrows show weak to null expression (scale bar, 100 µm). **C** MTT assay using adult rat primary SCs shows increased proliferation of cells in response to exogenous MANF supplementation (data presented as mean ± SE; One-Way ANOVA; Tukey’s multiple comparisons test; *n* = 5; *p < 0.05). **D** Brightfield images of scratch assay using adult rat primary SCs show a time-dependent gap closure in control and MANF (50 ng/ml) supplemented groups (scale bar, 100 µm). **E** Quantification of percentage gap closure in scratch assay shows increased gap closure in MANF (50 ng/ml) supplemented group compared to control (data presented as mean ± SE; Standard ‘t’ test; *n* = 3; *p < 0.05, **p < 0.01). **F** Schematic of transwell migration assay. **G** Dapi staining on the lower side of the membrane from a transwell migration assay shows migrated adult primary SCs (scale bar, 100 µm). **H** Quantification of cell migration in a transwell migration assay shows increased migration of primary SCs in MANF (50 ng/ml) supplemented group (data presented as mean ± SE; standard ‘t’ test; *n* = 3; **p < 0.01)

SCs are glial cells of the peripheral nervous system. They provide structural and trophic support to regenerating axons. Immediately after a peripheral nerve injury, SCs in the distal nerve stump de-differentiate, proliferate and then migrate towards the proximal nerve stump and guide the regenerating proximal axons toward target tissues. These properties of SCs are collectively referred to as ‘SC dynamics.’ Therapies designed to improve peripheral nerve regeneration should promote both axon regeneration and SC dynamics. Therefore, we examined the effect of MANF in SC dynamics using primary SCs isolated from adult rat sciatic nerves. The purity of the isolated primary SCs was confirmed using GFAP staining (Fig. 4A). We found a heterogeneous expression profile for MANF in these SCs, with some cells expressing MANF while others showing very low to null expression, which matches our previous observation that nerve-resident SCs express MANF only sparingly (Fig. 4B, 2E). MTT assay showed that MANF promotes the proliferation of primary SCs in a dose-dependent manner, with 100 ng/ml MANF inducing a significant increase in proliferation compared to control (Fig. 4C). Similarly, scratch assay showed improved gap closure after MANF supplementation, indicating that MANF promotes the migration of primary SCs (Fig. 4D, E). Improved gap closure in the scratch assay may also result from increased cell proliferation. Therefore, we performed a transwell migration assay to confirm the ability of MANF to promote SC migration (Fig. 4F). As expected, we observed increased migration of SC across the membrane, toward the MANF compartment, in the transwell assay, confirming the ability of MANF to promote SC migration by providing tropic signaling (Fig. 4G, H).

Next, we repeated the MTT and transwell migration assays in S16 SC line and found that supplementation of MANF promotes the proliferation and migration of these cells, too (Fig. S1). Overall, our results showed that MANF promotes SC dynamics, which is desirable for peripheral nerve regeneration.

### Local and repeated administration of MANF promotes axon regeneration in vivo

**Fig. 5:**
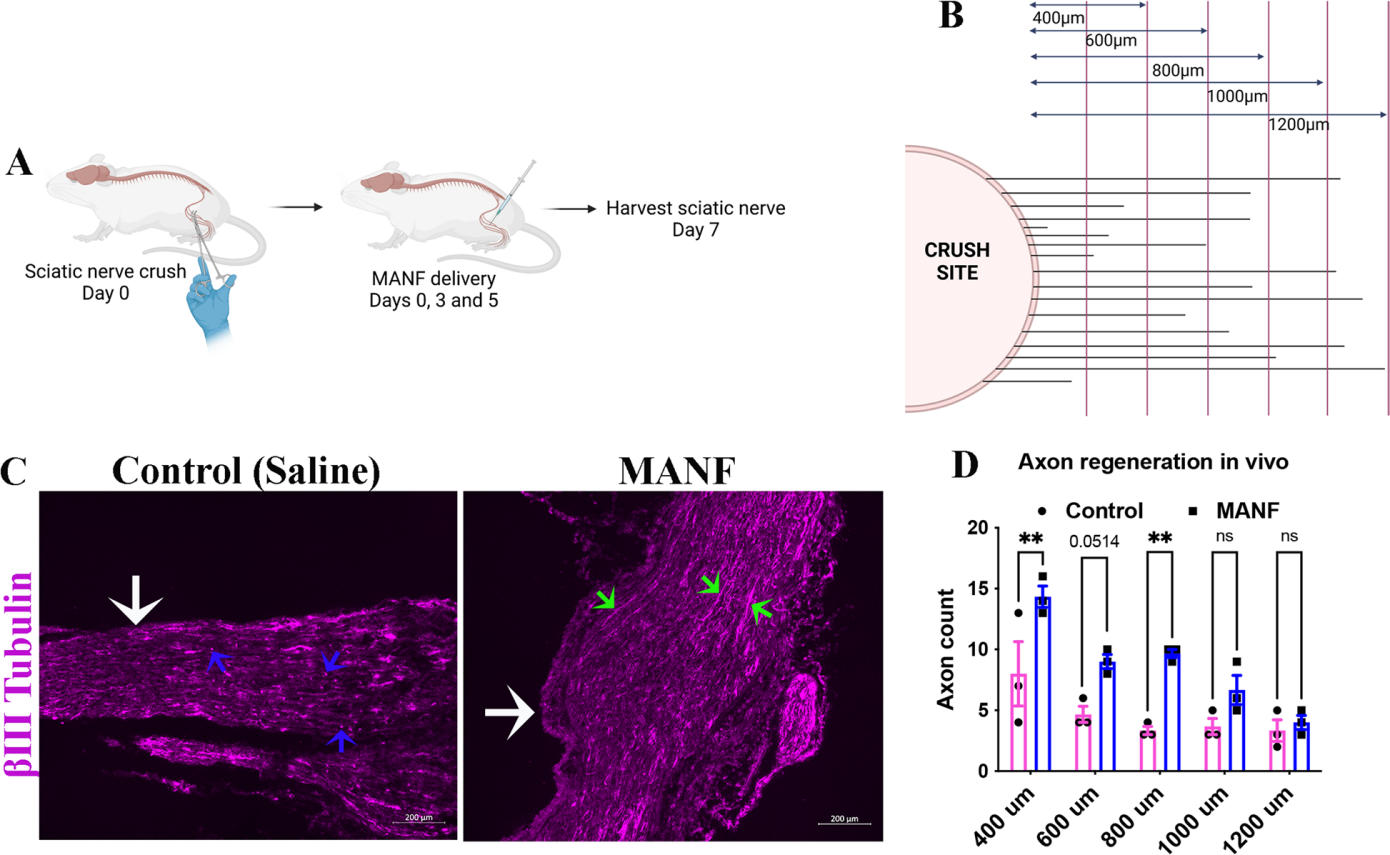
Local and repeated supplementation of MANF to injured nerve promotes axon regeneration. **A** Schematic of in vivo nerve crush injury and supplementation of MANF to nerves in adult mice. **B** Schematic of axon quantification approach. The number of axons crossing the perpendicular lines drawn at the shown distances from the crush site are manually counted to tabulate the axon count at each distance. **C** βIII tubulin staining of sections of injured sciatic nerves from adult mice shows degenerated (blue arrows) and intact (green arrows) axons in the control and MANF group, respectively. White arrows show the crush site (scale bar, 200 µm). **D** Quantification of intact axons in the distal nerve segment shows an increased number of intact axons in the MANF group compared to the control at varying distances from the crush site (data presented as mean ± SE; Two-Way ANOVA; Sidak’s multiple comparisons test; *n* = 3; **p < 0.01)

Effective nerve repair therapies, when seamlessly available to injured nerves, should promote axon regeneration. Therefore, to test if MANF delivered locally to injured nerves promotes axon regeneration, we directly injected MANF into adult CD1 mouse sciatic nerves after a crush injury. Crush injuries represent the most frequent type of nerve injuries presented to neuro-clinics. A crush injury induces axon degeneration in distal nerves, with partial or no disruption of connective tissue layers. We applied three doses of MANF (2 µg) at the site of injury, with the first dose given immediately after the injury, the second dose on day 3, and the third dose on day 5 (Fig. 5A). The nerves were harvested on day 7, and the regenerating axons in the distal nerve were quantified after βIII tubulin staining (Fig. 5B). We found an increased number of intact axons in the distal nerve in the MANF group compared to the control (saline), indicating that locally available MANF promotes axon regeneration in vivo (Fig. 5C, D). In contrast, the control group had more degenerating axon profiles observed in the distal nerve compared to the MANF group, suggesting that clearing of the degenerated axonal debris is also faster in the MANF group.

### Nerve resident SCs expressing Doxycycline (Dox)-inducible MANF protect axons and promote regeneration

**Fig. 6:**
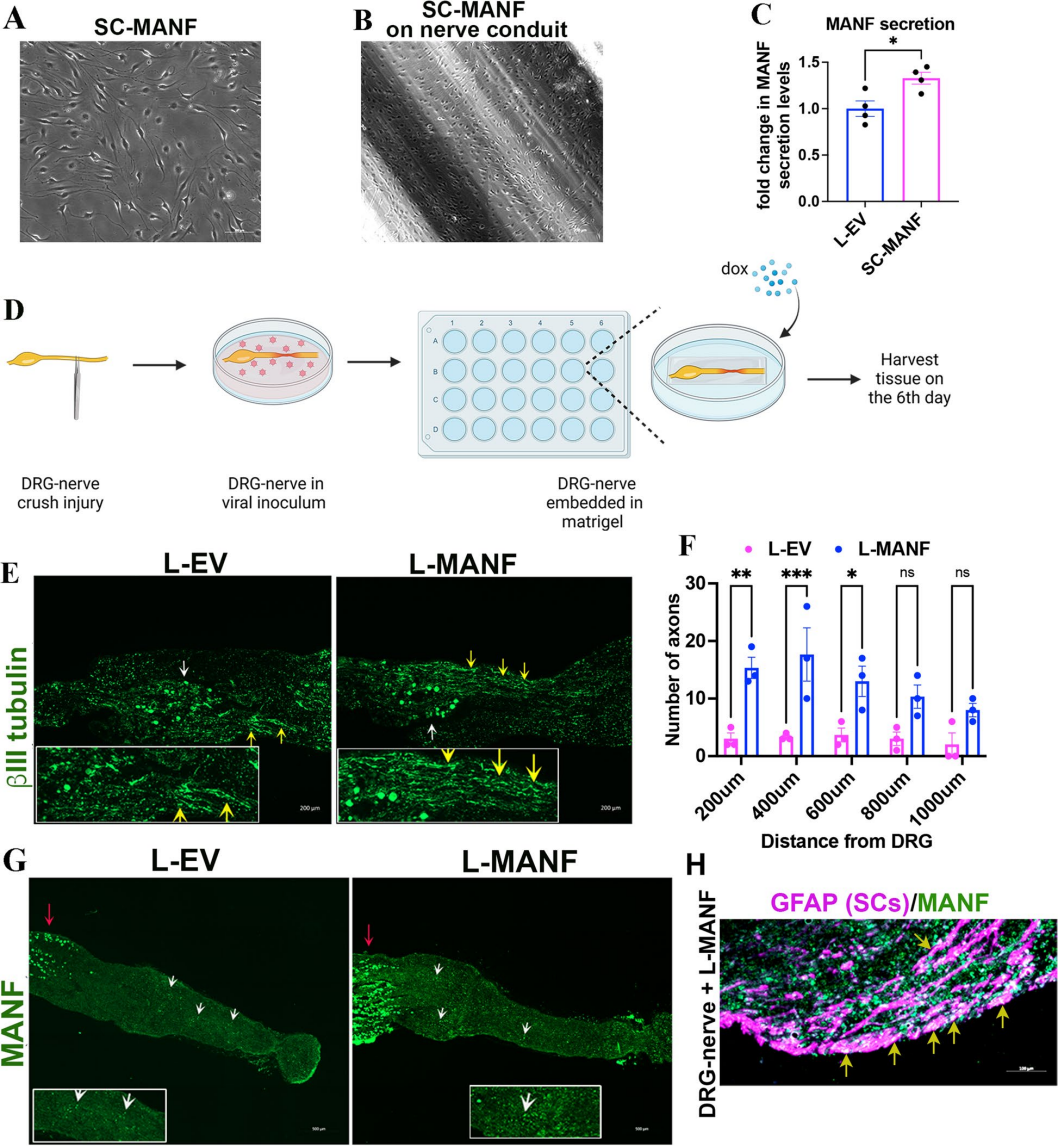
SC expressing Dox-inducible MANF (SC-MANF) protects axons and promotes regeneration. **A** SC-MANF generated using primary SCs after seven days of puromycin selection in a culture flask. **B** SC-MANF generated using primary SCs grows healthy on an artificial nerve conduit (15-day). (**C**) ELISA assay shows increased secretion of MANF by SC-MANF compared to L-EV transduced primary SCs after Dox supplementation (data presented as mean ± SE; standard ‘t’ test; *n* = 4; *p < 0.05). **D** Schematic of the nerve crush injury experiment using DRG-nerve explant. **E** Immunostaining of βIII tubulin in proximal sections of crush injured DRG-nerve explant on day 6. Yellow arrows show the presence of intact axons. The white arrow shows DRG (scale bar, 200 µm). **F** Quantification of intact proximal axons in the crushed DRG-nerve explants on day 6 shows an increased number of intact axons in the L-MANF group compared to the L-EV group (data presented as mean ± SE; Two-Way ANOVA; Sidak’s multiple comparisons test; *n* = 3; *p < 0.05, **p < 0.01, ***p < 0.001). **G** Immunostaining shows the expression of MANF in L-EV and L-MANF transduced DRG-nerve explants cultured in the presence of Dox (1 µg/ml). White arrows show the expression of MANF in the nerve segment, and the red arrow shows MANF expression in DRG (scale bar, 500 µm). Enlarged areas are provided in the insets. **H** A representative image shows the expression of MANF in SCs (yellow arrows-SC-MANF generation) in the nerve segment of a DRG-nerve explant transduced with L-MANF and cultured in the presence of Dox

Our findings above suggest that local and repeated supplementation of MANF promotes axon regeneration in vivo. However, surgical re-exposure of nerves for repeated delivery of MANF, as done for our proof-of-concept experiment shown in Fig. 5, is challenging for clinical practice. Therefore, we examined if nerve-resident SCs can be exploited as a local and sustained delivery system for MANF. To explore this possibility, we first expressed Dox-inducible MANF in cultured primary SCs using lentiviral transductions (Fig. S2). We found that the generated SC-MANF grows healthy on regular culture surfaces and an artificial nerve conduit (Fig. 6A, B). ELISA assay confirmed that SC-MANF secrete significantly higher levels of MANF as compared to lenti-empty vector (L-EV) transduced SCs following Dox exposure (Fig. 6C). We also confirmed the induction of MANF using western blot in SC-MANF generated using primary SCs and S16 (Fig. S3 A, B).

Next, to examine if nerve-resident SCs expressing Dox-inducible MANF (SC-MANF) promote axon regeneration, we used DRG-nerve explants isolated from adult SD rats. A nerve crush injury was made to the explants, and they were then transduced with either L-EV or L-MANF (to generate nerve-resident SC-MANF) and cultured in Cultrex extracellular membrane for six days in the presence of Dox (Fig. 6D). As opposed to nerve crushes in vivo, a nerve crush to DRG-nerve explant induces degeneration of proximal axons, especially during the first week of the injury. Interestingly, the L-MANF group showed an increased number of intact proximal axons on day 6 compared to the L-EV group (Fig. 6E, F). The greater number of intact axons in the L-MANF group may result from the combined neuroprotective and regenerative actions of MANF released from the induced SC-MANF in the explants. Immunostaining confirmed the increased induction of MANF in the L-MANF group compared to L-EV group after Dox exposure (Fig. 6G). We also confirmed the integration of L-MANF in nerve resident cells, including SCs *(generation of nerve-resident SC-MANF)*, throughout the nerve (Fig. 6H).

**Fig. 7:**
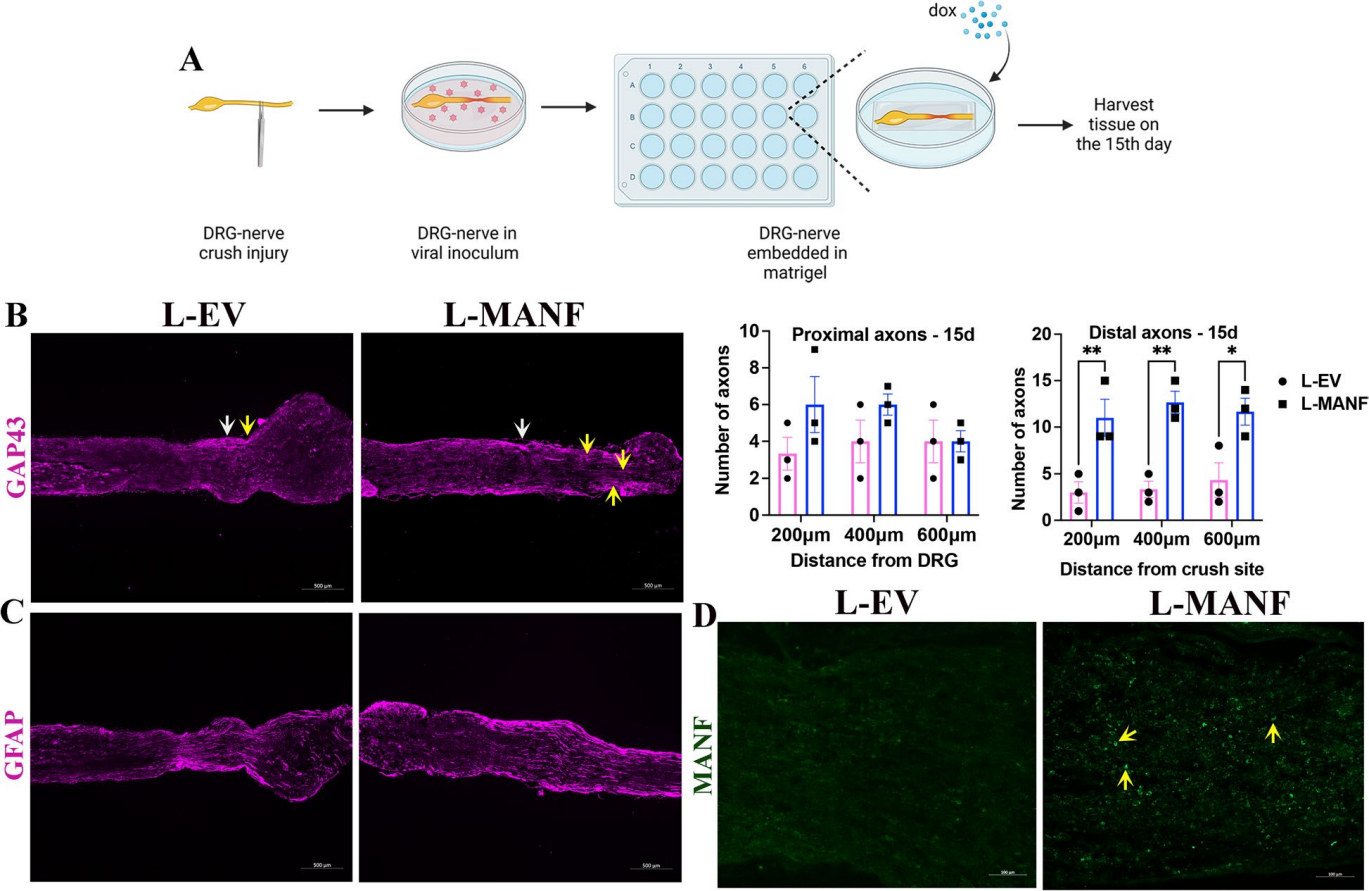
SC-MANF promotes axon regeneration. **A** Schematic and timeline of the nerve crush injury experiment using DRG-nerve explants. **B** (Left) Immunostaining of GAP43 in sections of DRG-nerve explants (day 15) shows longer regenerating axons (yellow arrows) past crush site (white arrow) in the L-MANF group compared the L-EV group (scale bar, 500 µm); (Right) Quantification of regenerating proximal (axons between DRG and crush site) and distal (axons past crush site) axons in DRG-nerve explants on day 15 shows a significant increase in regenerating distal axons in the L-MANF group compared to L-EV group (data presented as mean ± SE; Two-Way ANOVA; Sidak’s multiple comparisons test; *n* = 3; *p < 0.05, **p < 0.01). **C** Immunostaining of GFAP in sections of DRG-nerve explants (day 15) shows healthy distribution of SCs in the distal nerve (past crush site) (scale bar, 500 µm). **D** Immunostaining shows the expression of MANF (yellow arrows) in the nerve segments of L-EV and L-MANF transduced DRG-nerve explants cultured in the presence of Dox (1 µg/ml) for 15 days (scale bar, 100 µm)

We next repeated the explant experiment for 15 days to selectively evaluate regenerating axons crossing the crush site (Fig. 7A). GAP-43 staining showed the presence of a significantly increased number of regenerating axons in the distal nerves in the L-MANF group compared to the L-EV group, indicating significant axon regeneration (Fig. 7B). We also found an even distribution of SCs in nerves in the L-MANF group, indicating that SC integrity is maintained well (Fig. 7C). As observed in 6-day cultures, immunostaining of 15-day cultures showed an increased expression of MANF throughout the nerve in the L-MANF group compared to L-EV group after Dox exposure, confirming the induction of MANF in the L-MANF group (Fig. 7D, S3C, D). Collectively, these results show that nerve-resident SC-MANF is a potential cellular therapy for peripheral nerve regeneration.

## Discussion

Complete regeneration of peripheral nerves requires the coordinated actions of axons and SCs (Jessen and Mirsky 2016; Krishnan et al. 2024). Although such coordinated activities ensue for a few days to weeks following nerve injuries, the regenerating axons and SCs gradually lose mutual growth support, especially in the distal nerve segment, due to the poorly regrowing axons failing to re-establish contact with SCs. However, therapies that can improve and sustain the pace of axon growth and SC dynamics should be able to maintain the axon-SCs mutual support essential for complete regeneration. In this work, we demonstrated that MANF is such a potential therapy that can promote both axon growth and SC dynamics for nerve regeneration.

MANF is a neurotrophic factor (Petrova et al. 2003; Sivakumar and Krishnan, 2023). Previous studies showed that neurotrophic factors, such as nerve growth factor (NGF), can promote regeneration in animal models (Richardson 1991). However, translating NGF into a clinical therapy posed challenges because of its unexpected side effects and difficulties in maintaining its optimal levels in injured nerves (Alastra et al. 2021). NGF was also shown to induce pain, making it a less favorable choice for clinical therapy (Apfel et al. 2000). Although MANF is a neurotrophic factor, it is structurally and functionally distinct from the NGF family of neurotrophic factors (Lindholm and Saarma 2010). Also, NGF uses tropomyosin kinase receptor A (TrkA) for its growth promoting actions, while a specific receptor for MANF’s neurotrophic actions is unknown. The structural distinction of MANF to NGF points to a distinct growth promoting mechanism for MANF, independent of TrkA, raising its translational potential as a clinical therapy.

Human MANF is 100% conserved with rodent MANF, and this justifies the use of human recombinant MANF in our experiments. One reported distinction is, human MANF has an additional arginine-rich sequence (Sivakumar and Krishnan 2023)*.* While neurotrophic factors are attractive targets for nerve regeneration, their supraphysiological concentrations may suppress axon growth (Conti et al. 1997). In our experiments, we observed that 50 ng/ml of MANF improves outgrowth of injured neurons, while 100 ng/ml MANF was required to induce significant outgrowth in normal neurons. Injured neurons upregulate several neurotrophic factors compared to normal neurons, and this may be the reason why injured neurons respond to a comparatively lower concentration of MANF. This observation indeed suggests that properly controlling the local concentration of MANF may be critical for efficient nerve regeneration. We initially examined the effect of intraperitoneally administered MANF on nerve regeneration in a mouse model of sciatic nerve crush injury but did not find any improvement in regeneration and functional recovery (data not presented). However, local and repeated administration of recombinant MANF to injured nerves for a short period has improved axon regeneration in our experiments, suggesting that sustaining the local production of MANF in the regenerative milieu may offer therapeutic promise. However, repeated injections of MANF to nerves may not be deal for clinical practice as it poses technical challenges in addition to the challenges in controlling the precise quantity of the delivered MANF to nerves. Thus, harnessing SCs as a local delivery system for MANF, as explored in this study, becomes an attractive choice.

In addition to its neurotrophic actions, MANF modulates cellular stress by combating unfolded protein response (UPR) (Apostolou et al. 2008; Mizobuchi et al. 2007). The UPR buffering properties of MANF is linked to its neuroprotective actions (Wang et al. 2021). For example, experimental models of Alzheimer’s Disease, Parkinson’s Disease and stroke have demonstrated that MANF is a neuroprotector (Li et al. 2019; Liu et al. 2018; Xu et al. 2019). While our in vitro neurite outgrowth experiments showed that MANF promotes regeneration, the effect of MANF in preserving intact axons in our explant model may be attributed to its combined neuroprotective and growth promoting actions. Notably, our DRG-nerve explant experiment showed proximal axon degeneration in the control group in the first week period, while such degenerative events were comparatively lesser in the MANF group, indicating neuroprotection. At the same time, the 15-day DRG-nerve explant experiment showed longer distal regenerating axons in the L-MANF group compared to the control demonstrating the potential of MANF to improve nerve regeneration. Similarly, MANF promoted the axon growth past proximal stump in our in vivo crush injury experiment, further substantiating the neuroregenerative properties of MANF.

Our in vivo experiment was primarily focused on the effect of local MANF on nerve regeneration, and we found that local and repeated supplementation of recombinant MANF improves axon regeneration. A previous study demonstrated that decellularized human nerve grafts seeded with MANF-expressing mesenchymal stem cells improve nerve regeneration in adult rats (Lee et al. 2023). While we and this group used different injury models *(crush injury vs transection injury)* and MANF delivery approaches *(direct administration of MANF vs the use of allogenic mesenchymal stem cells-expressing MANF in decellularized human nerve graft),* both these studies showed that locally available MANF improves axon regeneration, substantiating that MANF is a potential neurotherapeutic. However, in this work, using in vitro experiments, we also demonstrated that exogenous MANF promotes SC dynamics critical for regeneration. This observation indeed revealed an opportunity for us to employ SCs as a carrier system for the local and sustained delivery of MANF. SCs are integral components of peripheral nerves, and their partnership with axons is critical for the proper functioning of nerves. Here we showed that SCs programmed to stably express MANF (SC-MANF) provide an excellent opportunity to timely and temporally deliver MANF to injured nerves. We also showed that SC-MANF protects axons and promotes regeneration. Importantly, the use of nerve-resident SC-MANF as a cellular therapy is comparatively safer than stem cell therapies, as autologous SCs carry no risk of immune rejection. However, further refinement of this approach is required, such as tailoring the Dox dosage regimen to accomplish the most optimal levels of MANF in regenerating nerves and evaluating this approach in chronic injury models, for the complete profiling of SC-MANF as a local therapy for peripheral nerve regeneration.

Overall, we demonstrated that MANF protects peripheral axons and induces a favorable growth response in both axons and SCs for peripheral nerve regeneration. We further showed that local supplementation of MANF to injured nerves at regular intervals promotes regeneration. We designed a potential cellular therapy for nerve regeneration by harnessing the capacity of SCs as a carrier system for the local and sustained delivery of MANF. Further refinement of this approach employing chronic injury models will offer an effective therapeutic choice for peripheral nerve regeneration.

## Materials and methods

### Sciatic nerve injury model

Four to six-week-old adult male SD rats were used for the sciatic nerve transection experiments, and 4- to 6-week-old adult male CD1 mice were used for in vivo sciatic nerve crush experiments. The animals were purchased from Charles River Laboratories, Canada. For nerve transection experiments, the sciatic nerve of rats was exposed, and a blunt transection made using fine scissors. Post operative analgesia was provided by administering sustained-release (SR) buprenorphine. Three days after the injury, the lumbar (L4, L5, and L6) DRGs were harvested from the ipsilateral side for individual neuron cultures, immunostaining, and western blot experiments. The proximal and distal nerve segments of the transected nerves were also harvested after three days of injury for western blot and immunostaining experiments.

For the nerve crush injury model, the sciatic nerve was exposed, and an injury was made by crushing the nerve using fine forceps for 15 s. MANF (2 µg/25 µl PBS) was applied immediately to the crushed site. Briefly, a parafilm was placed under the crushed nerve and the solution was injected to the nerve. While approximately 3~4 µl of solution (roughly equal to 400 ng) was contained in the nerve immediately following injection, the rest of the volume was overflown to the parafilm holding the nerve. We soaked the nerve in the overflown solution for 5 min before re-suturing the muscle and skin. The animals were maintained under analgesia with SR buprenorphine. On day 3 and day 5, the sciatic nerve was re-exposed for MANF treatment. Saline was applied to the control group. On day 7, the animals were euthanized. The sciatic nerves were harvested, fixed and immunostained for βIII tubulin for the quantification of axons using ImageJ.

### Immunohistochemistry

The DRGs and sciatic nerves were fixed in Zamboni’s buffer [2% paraformaldehyde (Cat No. F79-500, Fisher Scientific) and 0.5% picric acid (Cat No.5860–16, Ricca Chemical) in PBS] overnight at 4°C, followed by washed in PBS and incubated in 20% sucrose (Cat No. BP220-1, Fisher Scientific) for 24 h at 4°C. Tissue blocks were prepared using an optimal cutting temperature compound (Cat No. 23-730-571, Fisher Scientific) and 12 µm sections were collected onto slides using a cryostat. The sections were stored at −80°C until further analysis. For immunostaining, the sections were blocked using an immunoblocker [5% donkey serum (Cat No. 56-646-05ML, Fisher Scientific) and 0.3% Triton-X (Cat No. T8787-100ML, Sigma Aldrich) in PBS] for 40 min, washed in PBS and then incubated with primary antibodies for 1 h at RT. The primary antibodies used were MANF (Cat No. SAB3500384, Millipore Sigma, 1:100 dilution), NF200 (Cat No. N0142, Millipore Sigma, 1:100 dilution), βIII tubulin (Cat No. MAB1637, Millipore Sigma, 1:100 dilution), GFAP (Cat No. PA1-10,004, ThermoFisher Scientific, 1:100 dilution), and GAP43 (Cat No. PA5-143568, ThermoFisher Scientific, 1:100 dilution). The secondary antibodies used were goat anti-rabbit Alexa Fluor 546 (Cat No. A-11035, ThermoFisher Scientific, 1:100 dilution), goat anti-mouse Alexa Fluor 647 (Cat No. A-21235, ThermoFisher Scientific, 1:100 dilution), goat anti-rabbit Alexa Fluor 488 (Cat No. A-11034, ThermoFisher Scientific, 1:100 dilution), and goat anti-chicken Alexa Fluor 647 (Cat No. A-21449, ThermoFisher Scientific, 1:100 dilution). The secondary antibodies were incubated for 1 h at RT. For IB4 staining, the sections were initially incubated with Griffonia Simplicifolia Lectin 1 (GSL1) Isolectin B4 (IB4) (Cat No. L-1104-1, Vector Laboratories, 1:100) for 1 h at RT followed by incubation with goat anti-Griffonia Simplicifolia Lectin I antibody for 1 h at RT (Cat No. AS-2104-1, Vector Laboratories, 1:100 dilution). The sections were then developed using rabbit anti-goat Alexa Fluor 488 (Cat No, A-11078, ThermoFisher Scientific, 1:100 dilution). The sections were mounted using SlowFade Diamond Antifade mountant with DAPI (Cat No. S36973, ThermoFisher Scientific). The images were taken using a Zeiss Axio Observer inverted fluorescence microscope.

### Western blot

Proteins from tissues and cells were isolated using RIPA buffer (Cat No. PI8990, Fisher Scientific) in the presence of a halt protease and phosphatase inhibitor cocktail (Cat No. PI78441, MilliporeSigma). The protein concentration was determined using DC™ protein assay kit II (Cat No. 5000112, Bio-Rad) according to the manufacturer’s instructions. 20 μg of total protein was used for SDS-PAGE and the resolved proteins were transferred onto a PVDF membrane (Cat No. 1620177, Bio-Rad) using a semi-dry transfer method. The membrane was then blocked for 1 h in blocking buffer [5% skim milk powder and 0.1% Tween-20 (Cat No. BP337-500, Fisher Scientific) in TBS]. The primary antibodies used were MANF (Cat No. SAB3500384, Millipore Sigma, 1:1000 dilution) and GAPDH (Cat No. SAB3500247, Millipore Sigma, 1:1000 dilution) for overnight incubation at 4°C. The secondary antibodies used were goat anti-rabbit HRP (Cat No.1706515, Bio-Rad, 1:3000 dilution) and goat anti-chicken HRP (Cat No. A16054, ThermoFisher Scientific, 1:3000 dilution) for 1 h at RT. The blots were developed using an ECL kit (Cat No. 1705060, Bio-Rad) and imaged using a GelDoc (Bio-Rad). The quantification of band densities was done using ImageJ.

### Neurite outgrowth analysis

Primary sensory neurons were isolated from injured and normal DRGs, as described previously (Christie et al. 2014; Krishnan et al. 2021). Briefly, individual cells were enzymatically dissociated from DRGs by incubating the DRGs in 0.1% collagenase (Cat No. 17104019, ThermoFisher Scientific) for 90 min at 37 °C. Followed by the cells from the DRGs were mechanically dissociated by repeated manual pipetting. The resulted cell suspension was centrifuged at 800 rpm for 6 min. The cell pellet was suspended in L15 media and then layered over 15% bovine serum albumin (Cat No. SH3057402, Fisher Scientific) and centrifuged at 800 rpm for 6 min. The middle layer containing debris in the resulted supernatant was carefully removed, and the pellet was washed using L15 media. Finally, the pellet was resuspended in primary neuron culture media comprising DMEM/F12 (Cat No. 11-330-057, ThermoFisher Scientific), N2 Supplement (Cat No. 17502048, ThermoFisher Scientific, 1:100 dilution), 0.1% BSA (Cat No. SH3057402, ThermoFisher Scientific), 100 ng/ml NGF (Cat No. 13257-019, ThermoFisher Scientific) and antibiotic antimycotic solution (Cat No. SV3007901, Fisher Scientific, 1:100 dilution). An equal number of neurons were seeded into Nunc LabTek II 4-well Chamber Slide (Cat No. 154453, ThermoFisher Scientific) coated with 0.01% poly-l-lysine (Cat No. A005C, Fisher Scientific) and 10 μg/ml laminin (Cat No. 23017015, ThermoFisher Scientific). To study the growth-promoting effect of MANF, 50 ng/ml or 100 ng/ml recombinant human MANF (Cat No. 3748-MN-050, R&D Systems) was supplemented to neuron cultures. PBS was used as the control treatment. After 24 and 48 h, the cells were fixed using 4% paraformaldehyde (Cat No. P0018500G, ThermoFisher Scientific), blocked using immunoblocker [5% donkey serum (Cat No. 56-646-05ML) and 0.3% Triton-X (Cat No. T8787-100ML) in PBS] and immunostained for βIII tubulin (mouse monoclonal, Cat No. MAB1637, Millipore Sigma, 1:100 dilution) and NF200 (mouse monoclonal, Catalogue No. N0142, Millipore Sigma, 1:100 dilution). Goat anti-mouse Alexa Fluor 488 (Cat No. A-11001, ThermoFisher Scientific, 1:100 dilution) was used as the secondary antibody. Neurite images were captured using a Zeiss Axio Observer inverted fluorescent microscope. Quantification of neurite outgrowth was done using WIS-NeuroMath Software (Rishal et al. 2013).

### Primary SC cand S16 culture

Sciatic nerves were collected from adult male SD rats. The epineurium was stripped off using fine forceps, and the nerve teased out into individual fibres. The fibres were then triturated, suspended in 5 ml DMEM-D-Valine (Custom media, Boca Scientific) and centrifuged at 800 rpm for 5 min at 4 °C followed by incubation of the pellet in 1 mg/ml collagenase (Cat No. 17104019, ThermoFisher Scientific) for 60 min at 37°C. The fibres were then triturated using a flame-polished Pasteur pipette and incubated in 1% trypsin (Cat No. T4799-5G, Sigma-Aldrich) at 37°C for 30 min. The fibres were again triturated using a flame-polished glass pipette and passed through a 40 μm cell strainer (Cat No. 22-363-549, Fisher Scientific) into a falcon tube containing 2 ml of FBS. 25 ml of DMEM-D-valine was added to the filtrate and centrifuged for 5 min at 800 rpm at 4°C. The pellet was then resuspended in SC media and seeded into culture flasks coated with 0.01% poly-l-lysine (Cat No. A005C, Fisher Scientific) and 10 μg/ml laminin (Cat No. 23017015, ThermoFisher Scientific). The SC media was comprised of DMEM-D-Valine (Custom media, Boca Scientific), 10% FBS (Cat No.12483020, ThermoFisher Scientific), antibiotic antimycotic solution (Cat No. SV3007901, Fisher Scientific, 1:100 dilution), 2 mM glutamine (Catalogue #25030081, ThermoFisher Scientific), N2 supplement (Catalogue #17502048, ThermoFisher Scientific, 1:100 dilution), 10 μg/ml bovine pituitary extract (Cat No. 13028014, ThermoFisher Scientific), 5 mM forskolin (Cat No. F3917, Sigma-Aldrich) and 50 ng/ml heregulin (Cat No. 10003100 UG, Fisher Scientific).

The SC line S16 was purchased from the ATCC (USA). These cells were maintained in DMEM (Cat No. 30-2002, ATCC) containing 10% FBS (Cat No.12483020, ThermoFisher Scientific) and antibiotic antimycotic solution (Cat No. SV3007901, Fisher Scientific, 1:100 dilution). The cultures were maintained in a CO_2_ incubator at 37℃.

### Cell proliferation assay

SC proliferation was evaluated using MTT (3-(4,5 dimethylthiazol-2-yl)-2,5-Diphenyltetrazolium bromide) assay. Briefly, 5 × 10^3^ primary SCs or S16 cells were seeded into a 96-well plate previously coated with 0.01% poly-l-lysine and 10 μg/ml laminin. MANF was supplemented to these cells after 24 h. PBS was used as the control. After 24 h, the media was replaced with fresh media containing 500 μg/ml MTT (Cat No. M6494, ThermoFisher Scientific). After 4 h, the generated purple formazan crystals were dissolved in dimethyl sulfoxide (Cat No. BP231-1, Fisher Scientific). The absorbances were read at 570 nm (test) and 630 nm (background) using a SpectraMax M2 plate reader.

### Scratch assay

Scratch Assay was performed to evaluate the migration of SCs. 5 × 10^4^ primary SCs were seeded into cell culture plates previously coated with 0.01% poly-l-lysine and 10 μg/ml laminin. When the cells reached 90% confluency, a longitudinal scratch was made in the mid area of the plate using a p1000 pipette tip and the media was replaced with fresh media containing recombinant MANF. PBS was used as the control. Brightfield images of the scratch areas were taken at 0, 24 and 48 h time points using an Axio Observer inverted microscope. The areas of the scratches were measured using ImageJ software to evaluate the scratch closure and migration efficiency of SCs.

### Transwell migration assay

A transwell migration assay was performed to evaluate the migration of SCs in response to the tropic signals from MANF. Briefly, 5 × 10^4^ primary SCs or S16 cells were seeded into cellQART 24-well culture insert membranes (Cat No. 9318012, Sterlitech Corporation). The carrier plate was filled with media containing 50 ng/ml or 100 ng/ml MANF. After 48 h, the top surface of the membrane was scraped using a sterile cotton swab to remove the cells on the top surface. The insert was then washed with PBS. The membrane was then carefully cut out using a scalpel, inverted and mounted onto a Superfrost Microscopic Slide (Cat No. 12-550-15, Fisher Scientific) using DAPI-containing mounting media. Images of DAPI-positive cells were taken using an Axio Observer inverted microscope. The number of cells that migrated on to the lower surface of the membrane was counted using ImageJ.

### Preparation of lentivirus carrying Doxycycline (Dox)-inducible MANF construct

A codon optimized gene construct for human MANF was synthesized and cloned into pUC57 plasmid between EcoRI and NotI restriction sites by GenScript. The gene was excised from the pUC57 plasmid with EcoRI and NotI restriction enzymes and cloned into a Dox-inducible lentivirus plasmid pLVX TetOne Puro (Takara Bio) to generate the pLVX TetOne Puro MANF construct. The sequence of the plasmid was verified by nanopore-based sequencing with Plasmidsaurus. Lentiviruses carrying the MANF gene was produced by transducing HEK 293FT cells with pLVX TetOne Puro MANF plasmid along with the packaging plasmids pMD2.G and psPAX2 using polyethylenimine (PEI). Two days later, the cell culture supernatant containing the lentiviruses were harvested and stored at -80°C until use.

### Generation of SCs expressing Dox-inducible MANF

2 × 10^5^ primary SCs or S16 line were transduced with 100 μl of either lentivirus generated with pLVX TetOne Puro (lenti-empty vector) or pLVX TetOne Puro MANF (lenti-MANF) in the presence of 8 μg/ml polybrene (Cat No. TR-1003-G, Millipore Sigma). The cells were treated with 1 μg/ml puromycin (Cat No. P8833-25MG, Millipore Sigma) after 24 h to select stably transduced positive cell clones. Dox-inducible expression of MANF was evaluated in 80% confluent stably transduced cells after supplementing them with 1 μg/ml Dox (Cat No. AAJ6042203, ThermoFisher Scientific) for 24 h. The media was collected for ELISA assay to measure MANF secretion. Whole cell protein was isolated, and a western blot was performed to validate the overexpression of MANF.

### ELISA

ELISA was performed using the commercially available MANF ELISA Kit (Cat No. EKL58888-96T, Biomatik) according to the manufacturer’s instructions. Briefly, the standards and test samples were incubated in the wells for 90 min at 37℃. The solutions were then removed and 100 μl detection reagent A was added to the wells and incubated for 45 min at 37℃. Followed by the wells were washed with wash buffer for five times. After this step, 100 μl detection reagent B was added to the wells and incubated for 45 min at 37℃. The wash step was repeated and 90 μl of TMB substrate was added and incubated for 20 min at 37℃. Finally, a 50 μl stop solution was added to the wells, and the absorbance was read at 450 nm immediately.

### DRG-nerve explant crush injury model

Individual DRGs that maintain an intact connection with a 3~4 mm peripheral nerve was harvested from adult male SD rats. A crush injury was made in vitro to the nerve segment of the preparation approximately 2 mm distally to the DRG. The crushed DRG-nerves were then incubated with either lenti-empty vector or lenti-MANF in transduction media for 4 h in a CO_2_ incubator. Thereafter, the DRG-nerve preparations were embedded in Cultrex basement membrane extract (Cat No. 3433-010-01, R&D Systems) in a 24-well plate and cultured using standard primary neuron culture media supplemented with Dox (1 µg/ml). The tissue preparations were harvested on days 6 and 15. The 6-day timepoint explants were immunostained for βIII tubulin *(to assess neuroprotection)* and MANF, while the 15-day timepoint explants were immunostained for GAP43 *(to assess axon regeneration)*, GFAP, and MANF. The number of axons in the proximal *(from DRG to the crush site)* and distal *(from crush site to the distal end)* nerve segments of the explants was counted using ImageJ. For the quantification of the expression of MANF, the fluorescence counts of MANF were measured using the threshold method in ImageJ.

### Statistical analysis

The statistical analyses were performed using the GraphPad Prism 10 Software. The specific test performed, and the statistical significance achieved are presented in the figure legends.

## Supplementary Information

Supplementary Material 1: Figure S1. Exogenous MANF promotes SC dynamics. Figure S2. Maps of pLVX TetOne Puro (empty vector) and pLVX TetOne Puro MANF constructs. Figure S3. Expression of MANF by SC-MANF.

## Data Availability

The data generated and analyzed for this study are included in this article.

## Abbreviations

DRG: Dorsal Root Ganglion
ELISA: Enzyme-Linked Immunosorbent Assay
FBS: Fetal Bovin Serum
GAP43: Growth Associated protein 43
GAPDH: Glyceraldehyde-3-phosphate dehydrogenase
GFAP: Glial Fibrillary Acidic Protein
GSL 1: Griffonia Simplicifolia Lectin 1
HRP: Horse Radish Peroxidase
IB4: Isolectin B4
LC-MS: Liquid Chromatography- tandem Mass Spectrometry
L-EV: Lenti-empty vector
L-MANF: Lenti-MANF
MANF: Mesencephalic Astrocyte- Derived Neurotrophic Factor
MTT: 3-(4,5 dimethylthiazol-2-yl)-2,5-Diphenyltetrazolium bromide
NF200: Neurofilament 200
NGF: Nerve Growth Factor
PBS: Phosphate Buffered Saline
PVDF: Polyvinylidene Difluoride
RT: Room temperature
SC: Schwann Cells
SC-MANF: SCs expressing Dox-inducible MANF
SD Rats: Sprague-Dawley Rats
SDS-PAGE: Sodium Dodecyl Sulphate – Polyacrylamide Gel Electrophoresis
SR Buprenorphine: Sustained Release Buprenorphine
TBS: Tris-Buffered Saline
TMB: 3,3′,5,5′-Tetramethylbenzidine
TrkA: Tropomyosin kinase receptor A
UPR: Unfolded Protein Response
