# Supplementary material for: Schwann cells modified to secrete MANF is a potential cellular therapy for peripheral nerve regeneration
Source: Cell Regen. 2025 Jul 7;14:29. doi: 10.1186/s13619-025-00247-9 (PMC12234937; doi:10.1186/s13619-025-00247-9)
Supplement: Supplementary file 1 — Supplementary Material 1: Figure S1. Exogenous MANF promotes SC dynamics. Figure S2. Maps of pLVX TetOne Puro (empty vector) and pLVX TetOne Puro MANF constructs. Figure S3. Expression of MANF by SC-MANF. [file 13619_2025_247_MOESM1_ESM.docx]

**Supplementary figures**

**
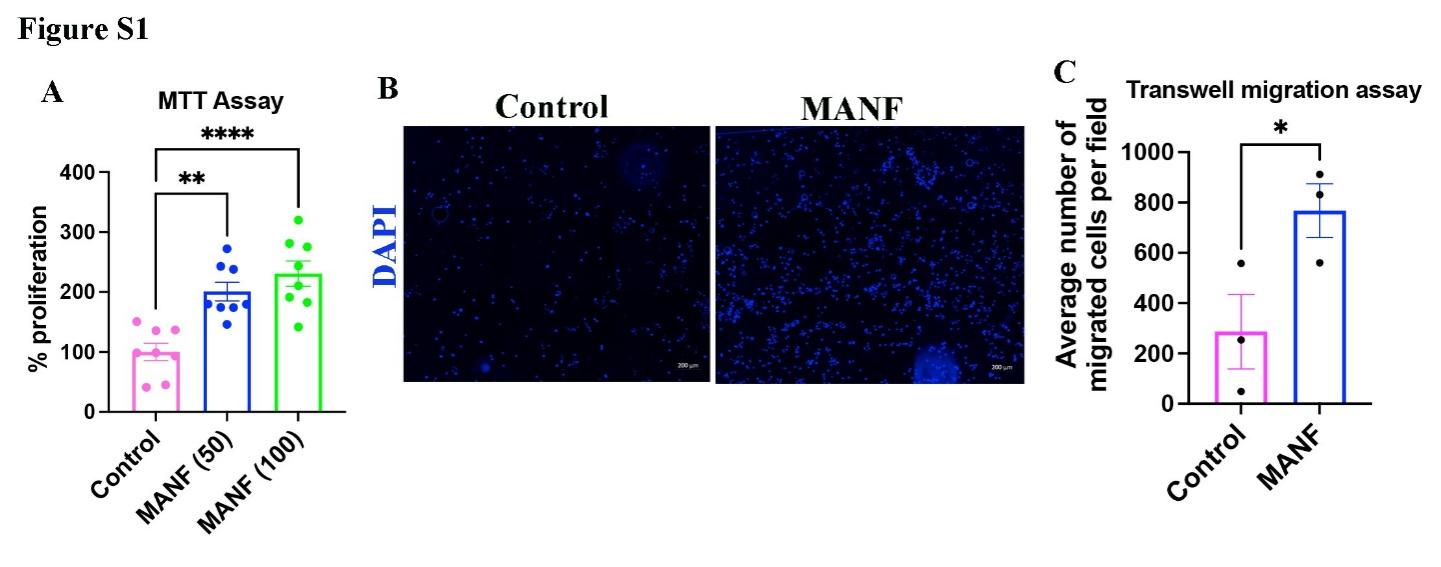
**

**Figure S1: Exogenous MANF promotes SC dynamics.** (A) MTT assay using S16 SC line shows increased proliferation of cells in response to exogenous MANF (data presented as mean ± SE; One-Way ANOVA; Tukey’s multiple comparisons test; n=8; **p<0.01, ****p<0.0001). (B) Dapi staining on the lower side of the membrane from a transwell migration assay shows migrated S16 SCs at 48h in the control and MANF group (scale bar, 200µm). (C) Quantification of SC migration in the transwell assay shows increased migration of S16 SCs in MANF (100 ng/ml) supplemented group at 48h (data presented as mean ± SE; standard ‘t’ test; n=3; *p<0.05)

**
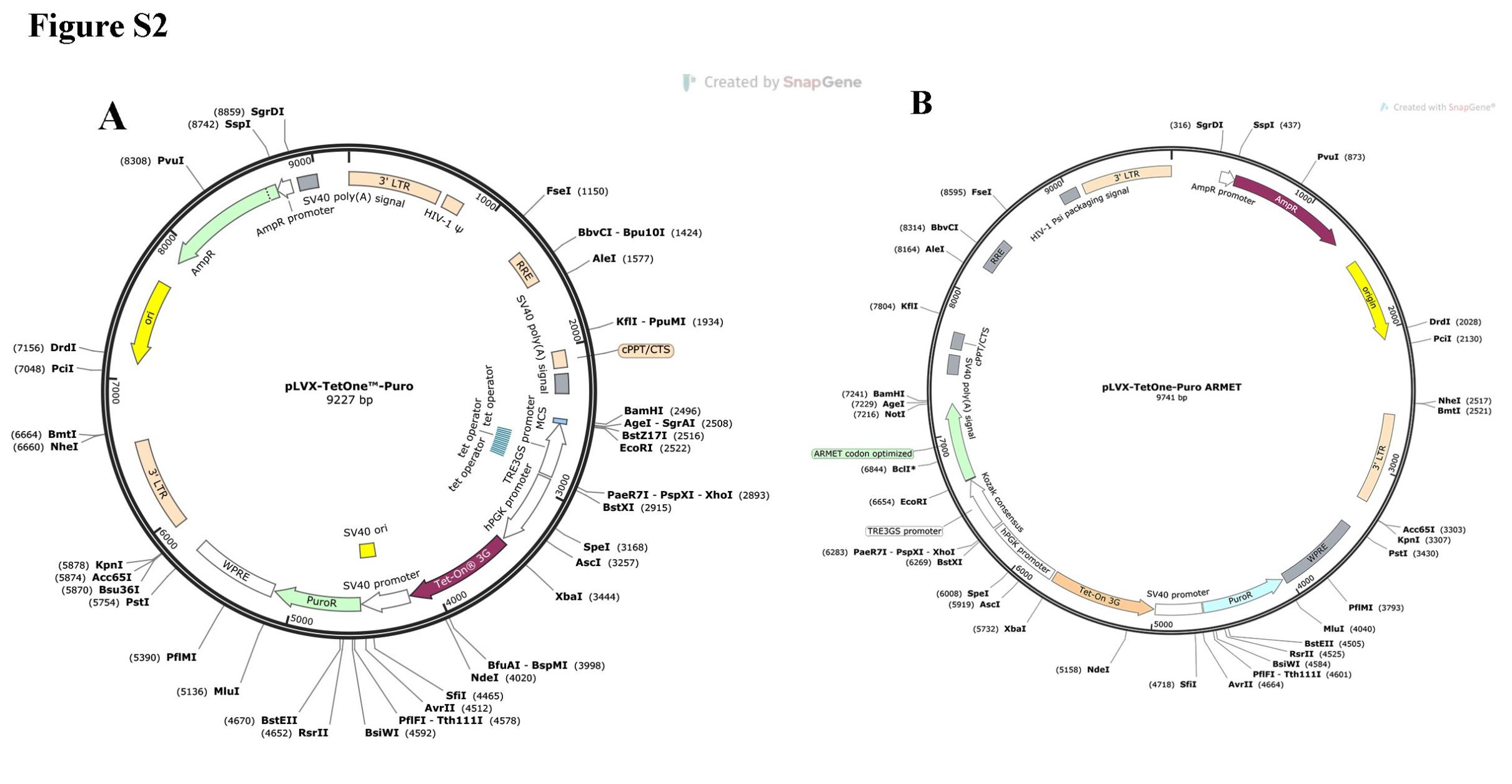
Figure S2:** Maps of (A) pLVX TetOne Puro (empty vector) and (B) pLVX TetOne Puro MANF constructs.

**
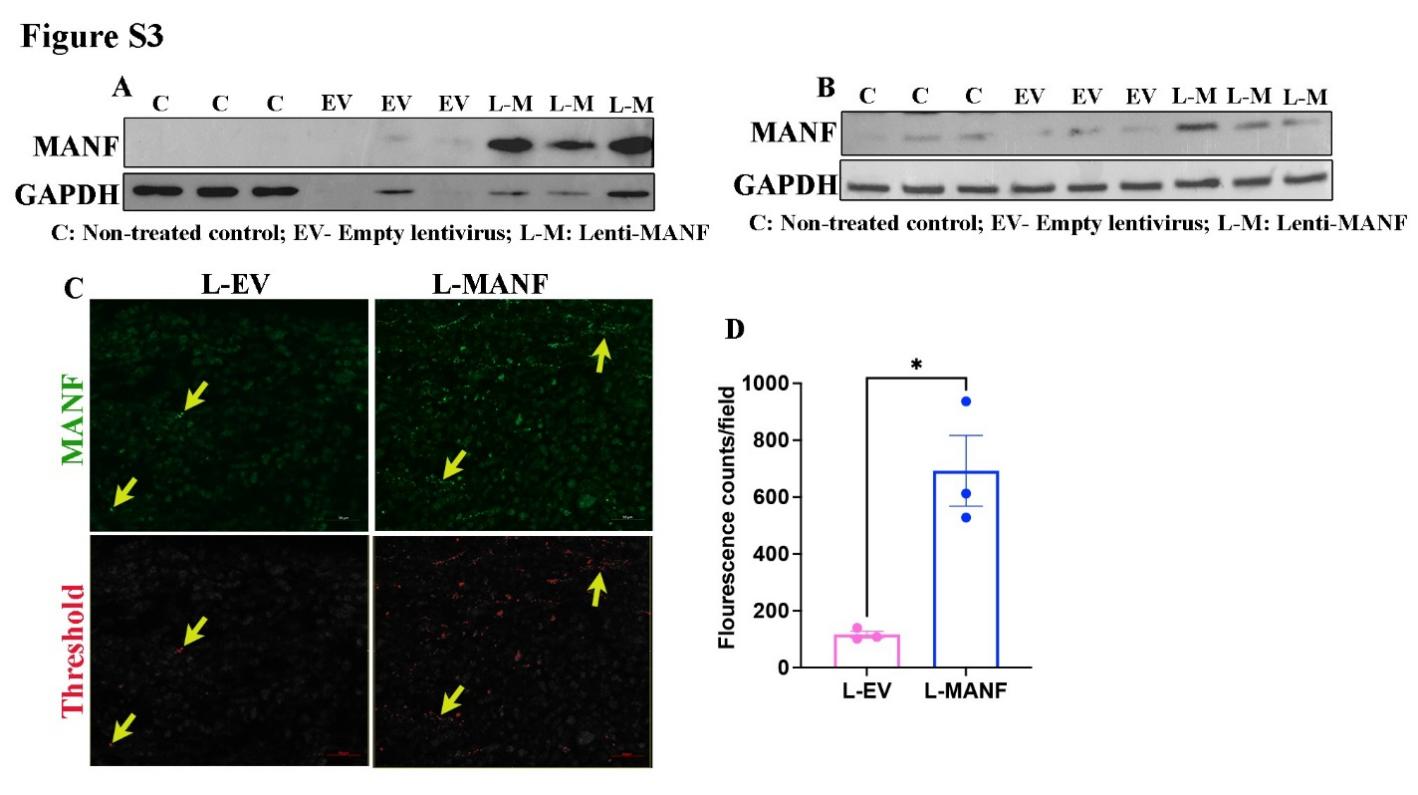
**

**Figure S3: Expression of MANF by SC-MANF.** (A) Western blot shows the expression of MANF in untreated, L-EV transduced, and L-MANF transduced primary SCs after Dox exposure. GAPDH is used as the loading control. (B) Western blot shows the expression of MANF in untreated, L-EV transduced, and L-MANF transduced S16 after Dox exposure. GAPDH is used as the loading control. (C) (top) Representative immunostaining images of MANF in L-EV and L-MANF treated DRG-nerve explants grown in Dox supplemented media for 15 days showing MANF expression (yellow arrows); (bottom) Corresponding ImageJ thresholded images for fluorescence count quantification. (D) Quantification of MANF fluorescence counts in L-EV and L-MANF treated DRG-nerve explants grown in Dox supplemented media for 15 days shows increased MANF expression in the L-MANF group (data presented as mean ± SE; standard ‘t’ test; n=3; *p<0.05).
